# Down‐regulation of ER‐α36 mRNA in serum exosomes of the patients with hepatocellular carcinoma

**DOI:** 10.1002/ctm2.18

**Published:** 2020-05-13

**Authors:** Hui Huang, Zhiyuan Zhou, Hongyan Li, Yong Zhang, Liang Zhao, Zhidong Wang, Qiqi Zhang, Chunyan Liu, Changxin Han, Qi Wang, Chunwen Pu, Wei Zou

**Affiliations:** ^1^ Department of Biobank The Affiliated Sixth People's Hospital of Dalian Medical University Dalian China; ^2^ College of Life Science Liaoning Normal University Dalian China

Epidemiological data indicated that the incidence and mortality of hepatocellular carcinoma (HCC) in males are higher than that in females.^1^ In 2018, the ratio of the number of cases to deaths in men and women was about 2.5:1 and 2.3:1, respectively. However, the incidence of HCC in postmenopausal women is significantly higher, which is almost equal to the incidence of men,^2^ suggesting that the estrogen‐signaling pathways play a vital role in development of HCC. The estrogen receptor alpha 66 (ER‐α66), commonly used as a breast cancer marker, is a well‐known mitogenic nuclear receptor.^3^ It has been reported that ER‐α66 is involved in the development of HCC.^4^ However, the role of ER‐α66 in the diagnosis of HCC is uncertain. The estrogen receptor alpha 36 (ER‐α36) is a novel estrogen receptor variant discovered by Wang et al in 2005.^5^ Studies have shown that ER‐α36 mediates the non‐genomic effects of estrogen and participates in the progress of different cancers through MAPK/ERK, PI3K/Akt, and other signaling pathways.^6^, ^7^ Studies have also shown that the signal‐regulating loop formed by ER‐α36 and EGFR can affect the proliferation of HCC,^8^ but its role in the diagnosis of HCC has not been reported before.

In recent years, exosomes have attracted widespread attention as a new pathway for intercellular communication. Exosomes are membrane‐like vesicle structures with a diameter of 30‐150 nm.^9^ Almost all cells in the human body release exosomes under physiological conditions, and tumor cells are a generous producer of exosomes that carry a variety of genetic and molecular cargoes that reflect the parental cells. A large number of studies have shown that tumor‐derived exosomes play an important role in tumor proliferation, invasion, metastasis, angiogenesis, and drug resistance.^10^, ^11^ In addition, bioactive molecules in exosomes can be used for the diagnosis and prognosis of cancer.^12^, ^13^ However, the clinical significance of estrogen and its receptor in HCC exosomes is unknown. Our previous study has found that the combined examination of extracellular miR‐21 and miR‐144 in serum is helpful in diagnosing HCC,^14^ suggesting that extracellular vesicles or exosomes may provide novel approaches for the diagnosis and prognosis of HCC.

Thus, we decided to examine the expression of estrogen and its receptor variants, ER‐α66 and ER‐α36, in HCC tissue and blood samples. Here, we report our study on the expression pattern of estrogen and its receptor variants in serum exosomes of HCC, and provide insightful information for the diagnosis of human HCC.

Two hundred and thirty people meeting the experimental criteria were recruited from January 2015 to January 2018 at the Affiliated Sixth People's Hospital of Dalian Medical University. The people were categorized in the following four categories: normal, chronic liver disease (CHB), liver cirrhosis (LC), and hepatocellular carcinoma (HCC). CHB, LC, and HCC were all diagnosed according to the standards of the Asian Pacific Association for the Study of the Liver, and normal category was a healthy donor with no‐disease detected. The study was approved by the ethics committee of the Affiliated Sixth People's Hospital of Dalian Medical University (2016‐013‐007). The tissue and blood samples used in the study were obtained with the patient's informed consent.

First, the surgically resected HCC tissue and its adjacent normal tissue were selected for IF staining (primary antibody: ER‐α66, 1:400, CST, 13258; ER‐α36, 1:300; fluorescence‐conjugated secondary antibody: 1:400, Jackson, 111‐165‐003, and 115‐605‐003). The results showed that ER‐α66 and ER‐α36 were expressed in both HCC and adjacent normal tissue, and located in the cytoplasm and cell membrane of hepatocytes and HCC cells (Figure 1A). Second, the levels of E2 in plasma from the patients with CHB, LC, and HCC were examined to determine the changes of E2 in blood during the process of CHB developing into HCC (patient clinical data in Table 1) with the Access Estradiol Kit (Beckman Coulter, 33540). In order to avoid the female cyclical changes affect estrogen levels in blood, only male patients were recruited in this experiment. Results showed that plasma estrogen levels were found to be significantly elevated in LC patients compared with that in CHB patients, and significantly lower in the HCC patients compared with the LC patients (*P* < .05) (Figure 1B), suggesting that estrogen may be involved in the regulation of the development of HCC.

**FIGURE 1:**
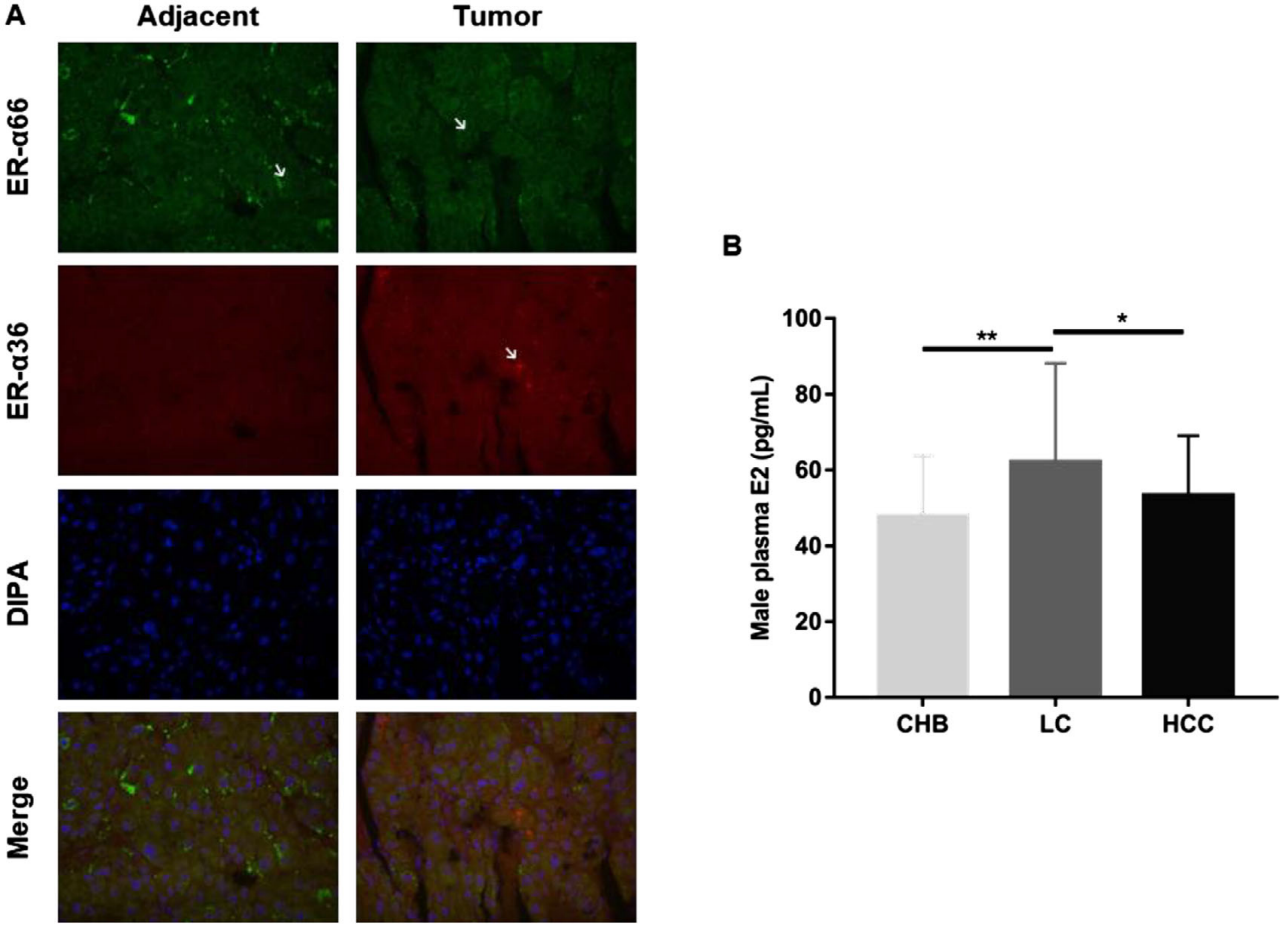
The expression of estrogen and estrogen receptor variants (ER‐α66 and ER‐α36) in hepatocellular carcinoma (HCC). A, IF staining of ER‐α66 and ER‐α36 in HCC and adjacent tissue (×400). B, Expression of estrogen in HCC (male): chronic liver disease (CHB, 47.98 ± 15.76, n = 55), liver cirrhosis (LC, 62.14 ± 26.06, n = 56), and HCC (53.32 ± 15.72, n = 56) patients with plasma E2 levels (*P < .05, **P < .01)

**TABLE 1:** Patient cohort description of 17β‐estradiol

Clinical parameter		CHB (n = 55)	LC (n = 56)	HCC (n = 56)
Age (years)	<60	51	43	37
	≥60	4	13	19
Gender	Male	55	56	56
	Female	0	0	0
HBsAg	Positive	55	49	40
	Negative	0	5	1
AFP (ng/mL)	<400	52	54	35
	≥400	1	0	17
ALT (U/L)		162.27 ± 205.36	53.77 ± 76.59	55.28 ± 53.93

Second, we detected the expression patterns of ER‐α66 and ER‐α36 using IHC kit (Universal Two‐Step IHC Kit, ZSGB‐BIO, PV‐9000) with antibodies against ER‐α66, 1:100, Abcam, ab3575 and ER‐α36, 1:100, and Western blot with antibodies for ER‐α66, 1:5000, CST, 13258; ER‐α36, 1:5000; HRP‐conjugated secondary antibody: 1:5000, Abcam, ab6789 and ab6721 (patient clinical data in Table 2). The results of IHC showed that ER‐α66 expression was significantly downregulated in HCC tissue compared with adjacent tissue in 73.1% (19/26) patients (*P* < .05). The expression of ER‐α36 was down‐regulated in HCC tissue in 50% (5/10) patients (Figure 2A‐C). Compared with adjacent tissue, the proportion of patients with ER‐α66 and ER‐α36 down‐regulated in HCC tissue was 66.7% (4/6), consistent with the results of IHC (Figure 2D‐F).

**TABLE 2:** Patient cohort description of hepatocellular carcinoma (HCC)

Clinical parameter		HCC (n = 26)
Age (years)	<60	12
	≥60	14
Gender	Male	19
	Female	7
HBsAg	Positive	19
	Negative	7
AFP (ng/mL)	<400	20
	≥400	6
CA19‐9 (U/mL)		17.79 ± 14.35
CEA (ng/mL)		2.97 ± 2.21
ALT (U/L)		36.12 ± 21.82
AST (U/L)		33.78 ± 15.33
GTD (cm)	<3	16
	≥3	10

**FIGURE 2:**
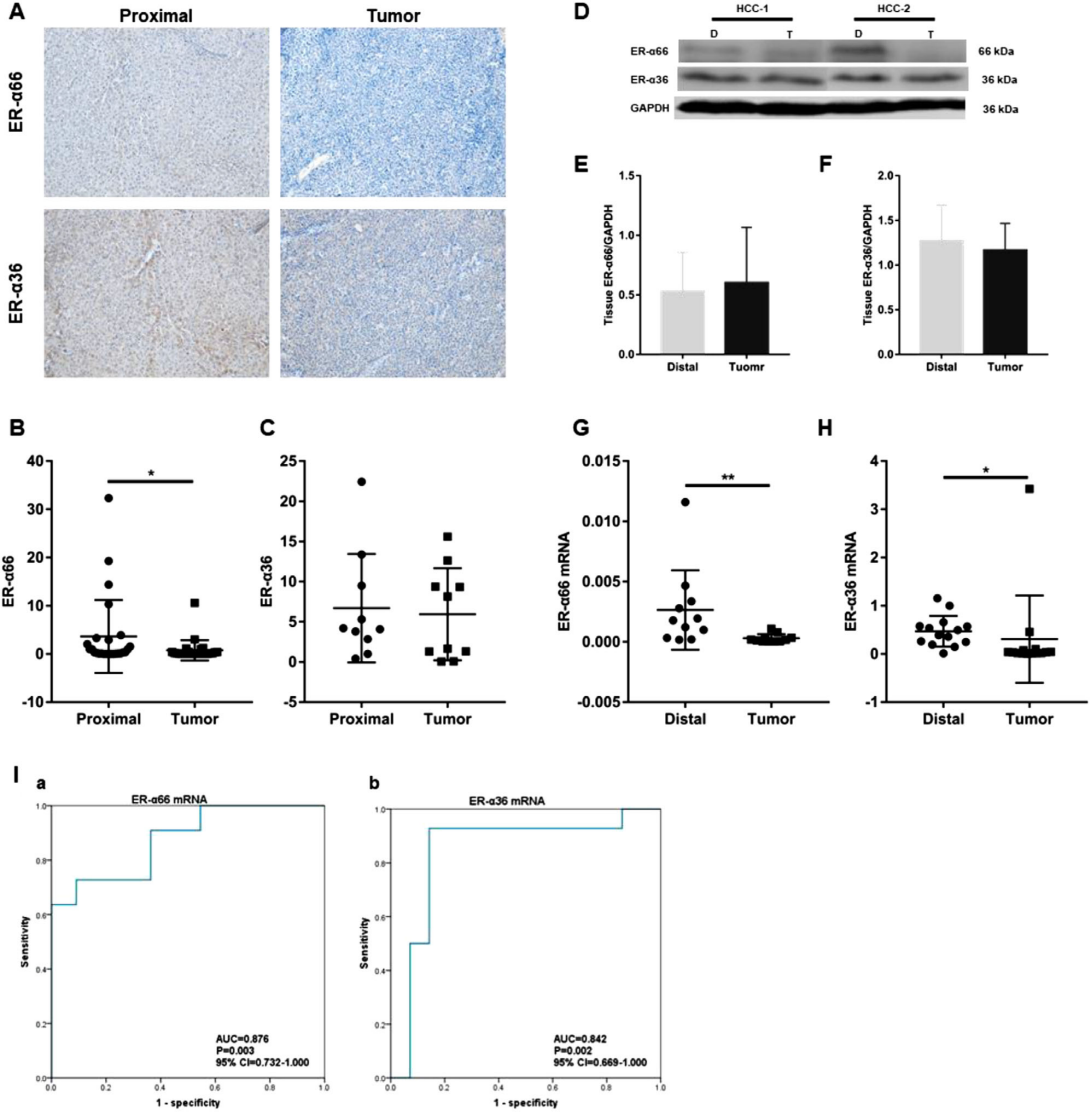
ER‐α66 and ER‐α36 were both down‐regulated in hepatocellular carcinoma (HCC) tissue. A, IHC staining of ER‐α66 and ER‐α36 proteins in HCC and adjacent tissues; ER‐α66 and ER‐α36 were mainly located in the cytoplasm and cell membrane (×400). B and C, Quantification of IHC results for ER‐α66 (adjacent: 3.62 ± 7.57 and tumor: 0.74 ± 2.12, n = 26) and ER‐α36 (adjacent: 6.69 ± 6.75 and tumor: 5.94 ± 5.73, n = 10). D, Western blot of ER‐α66 and ER‐α36 in HCC and adjacent tissues. E and F, Quantification of Western blot results for ER‐α66 (adjacent: 0.53 ± 0.33 and tumor: 0.60 ± 0.47, n = 6) and ER‐α36 (adjacent: 1.27 ± 0.40 and tumor: 1.16 ± 0.30, n = 6). G and H, qRT‐PCR results of ER‐α66 mRNA (adjacent: 0.0026 ± 0.0033 and tumor: 0.0003 ± 0.0003, n = 11) and ER‐α36 mRNA (adjacent: 0.4700 ± 0.3180 and tumor: 0.3058 ± 0.9045, n = 14) with *P < .05 and **P < .01, respectively. I, Efficiency of ER‐α66 and ER‐α36 mRNA in the diagnosis of HCC: (a) ROC curves of ER‐α66 mRNA; (b) ROC curves of ER‐α36 mRNA

Finally, the expression of two variants of ER‐α in HCC and adjacent tissue was further examined at mRNA level. Tissue mRNA was extracted with miRcute miRNA Isolation Kit, (TANGEN, DP501); reverse transcribed (FastKing RT Kit, TIANGEN, KR116) and examined with qRT‐PCR assay (SuperReal PreMix Plus, TIANGEN, FP205, the primer sequences are included in the Supporting Information Table). The mRNA levels agreed well with the protein levels. Compared with adjacent tissue, the mRNA levels of ER‐α66 (*P* < .001, Figure 2G) and ER‐α36 (*P* < .05, Figure 2H) were significantly down‐regulated in HCC tissue. In addition, the ROC curve was used to analyze the possibility of using two ER‐α mRNAs for the diagnosis of HCC. The results showed the area under the curve (AUC) of ER‐α66 mRNA was 0.876 (*P* = .003, 95% CI = 0.732‐1.000) and that of ER‐α36 mRNA was 0.842 (*P* = .002, 95% CI 0.669‐1.000), indicating that mRNAs of ER‐α66 and ER‐α36 show good accuracy for the diagnostic of HCC and could be effectively used to distinguish HCC from adjacent tissue (Figure 2I).

In further experiments, exosomes in serum of the patients with HCC were isolated and identified using Exo‐spin Exosome Purification Kit (Cell Guidance Systems, EX02) to examine the expression pattern and clinical significance of ER‐α36 in exosomes. Transmission electron microscopy and nano‐particle size concentration analysis (ZetaView PMX 110, Particle Metrix, Meerbusch, Germany; software: ZetaView 8.04.02) showed that the obtained samples were concentrated in the diameter between 30 and 150 nm, and the concentration was above 3 × 10^6^, which agrees well with exosome characteristics (Figure 3A). In addition, serum exosomes from normal, and the patients with CHB and HCC were collected, and then ER‐α36 expression was examined using Western blot analysis. Western blot results showed that ER‐α36 was present at the lowest level in the exosome from the normal group and at the highest from CHB group (Figure 3B), and the qRT‐PCR results showed that the mRNA expression of ER‐α36 was significantly down‐regulated in HCC group (*P* < .01) compared to CHB group, consistent with the trend found in the Western blot results (Figure 3C). The ROC curve showed that the ER‐α36 mRNA in the exosome could effectively distinguish the patients with CHB and HCC (AUC = 0.828, *P* = .005, 95% CI = 0.675‐0.980, Figure 3D), suggesting that ER‐α36 mRNA in serum exosomes potentially has a role in HCC diagnosis.

**FIGURE 3:**
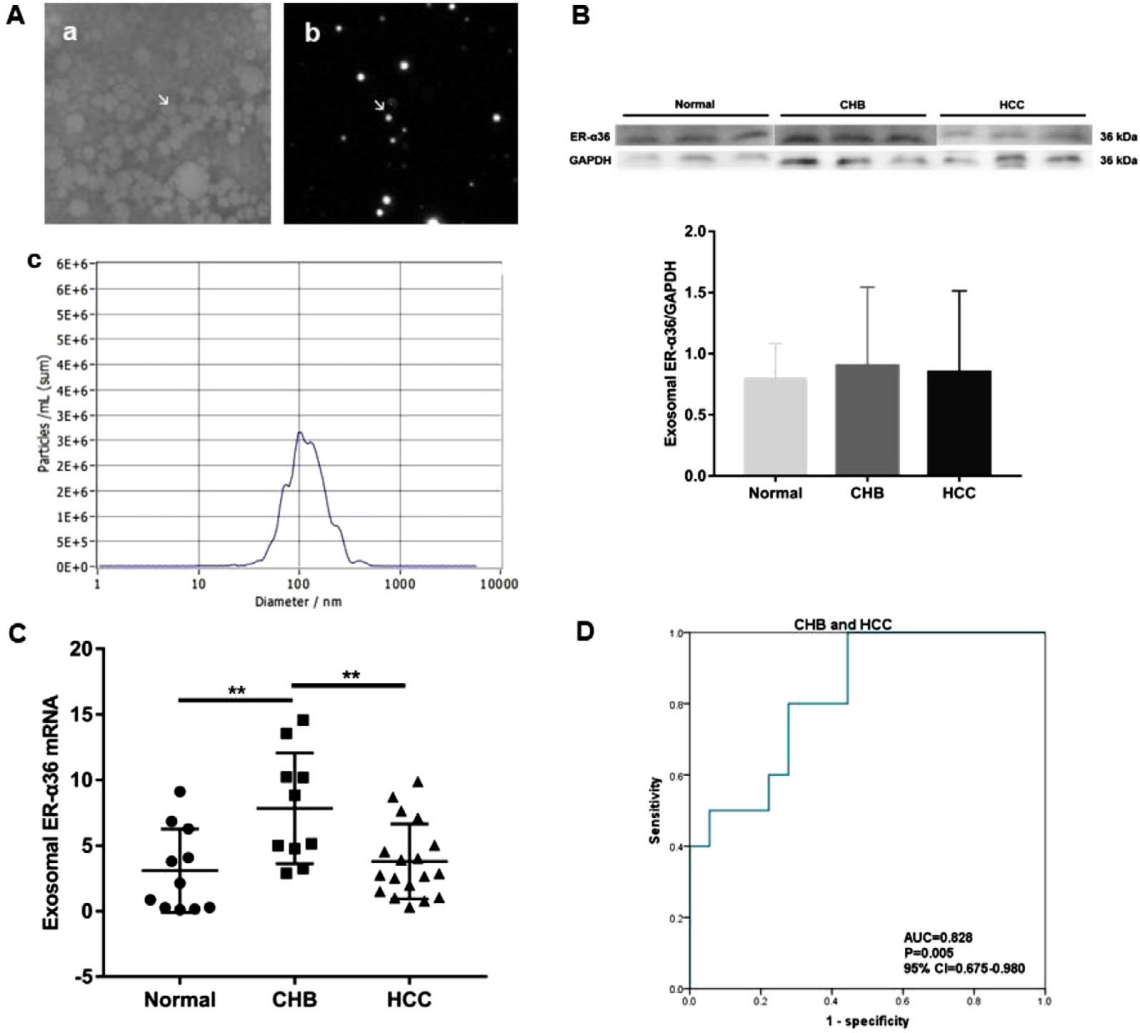
ER‐α36 mRNA was significantly down‐regulated in the exosome from the HCC patients. A, Identification and characterization of serum exosomes from the patients with HCC: (a) Transmission electron micrograph of purified exosome (30‐110 nm in diameter; bars 100 nm; arrow indicates exosome); (b) Morphology of exosome using ZetaView; (c) Particle size and distribution of exosome using ZetaView. B, Western blot quantification of ER‐α36 in the serum exosomes from the normal subjects (0.80 ± 0.28, n = 6), and the patients with CHB (0.90 ± 0.64, n = 6) and HCC (0.85 ± 0.66, n = 6). C, qRT‐PCR result of ER‐α36 in the serum exosomes from the normal subjects (3.09 ± 3.18, n = 11), and the patients with CHB (7.84 ± 4.23, n = 10) and HCC (3.79 ± 2.86, n = 18) (**P < .01). D, ROC curve analysis of ER‐α36 mRNA in the exosomes from the patients with CHB and HCC

Estrogen and its receptors are involved in the development and progression of breast cancer, but their expression pattern and clinical significance in the serum exosomes of HCC are not known. There is evidence indicating that estrogen may be a protective factor against the progression of liver cancer in the patients with HBV infection.^15^ In this paper, we found that the plasma estrogen levels were significantly elevated in the LC patients compared with that in the CHB patients, and significantly lower in the HCC patients compared with the LC patients. Serin et al reported that liver estrogen inactivation capacity in the patients with cirrhosis is weakened, leading to an increase in blood estrogen levels.^16^ This finding is consistent with our results, suggesting that estrogen may be involved in the development of HCC.

ER‐α (ESR1) and ER‐β (ESR2) are two major subtypes of estrogen receptor. Changes in the structure and content of these two subtypes will eventually lead to changes in the physiological functions of estrogen. Most of the biological effects of estrogen in the liver are mediated by ER‐α.^17^ Abnormal expression of ER‐α in the liver is associated with hepatocyte proliferation, which may induce or promote liver diseases.^18^ Furthermore, in the early stages of CHB, the variants of ER‐α are highly expressed in male patients compared with female patients, and are overexpressed in tumor tissues compared with normal tissues.^19^, ^20^ Therefore, the variants of ER‐α may have diagnostic value in liver cancer.

ER‐α66 is the classical estrogen receptor that mediates the genomic effects of estrogen. At present, ER‐α66 has been widely used as a marker for the diagnosis and treatment of breast cancer,^3^ but its diagnostic value in HCC is unknown. In this study, we found that ER‐α66 expression in most HCC tissue was lower than that of the adjacent tissue, and the ROC curve showed that ER‐α66 mRNA had a good diagnostic value in HCC. Miceli et al found that ER‐α66 is highly expressed in normal liver tissue, but is almost absent in HCC tissue,^21^ consistent with our results. Meanwhile, Wang et al reported that both mRNA and protein levels of ER‐α66 were downregulated in HCC HepG2 cells (*P* < .01), and the ER‐α66 mRNA levels in the tumor tissue were up‐regulated compared with the non‐cancerous tissues (*P* < .05).^22^ In fact, we also observed an up‐regulation of ER‐α66 in a small subset of HCC samples, the underlying mechanism of which requires further research.

ER‐α36 is a novel variant of ER‐α66 discovered by Wang et al in 2005.^5^ Previous studies have found that high ER‐α36 expression is often accompanied by a high degree of malignancy and poor prognosis of cancer,^22^ indicating that ER‐α36 plays an important role in tumor progression. Studies have shown that ER‐α36 is upregulated in HCC tissue and is more expressed in primary liver cancer than in secondary liver cancer.^23^ In this study, we found that ER‐α36 expression in the HCC tissues from most patients was lower than that of the adjacent tissues, and the ROC curve showed that ER‐α36 mRNA in cancer tissues has diagnostic value in HCC. However, ER‐α36 expression in a small portion of liver cancer samples was found increased compared to adjacent tissues, the exact reason of which requires further investigation.

Exosomes are carriers of biologically active substances, and are ideal materials for liquid biopsy. However, there are few reports that described mRNA in exosomes for the diagnosis of human HCC. In this study, we discovered that ER‐α36 was present in the exosome of the patients with HCC and ER‐α36 mRNA was significantly downregulated in exosome from HCC patients. In addition, the ROC curve showed that ER‐α36 mRNA in exosomes could effectively distinguish the patients with CHB and HCC, suggesting that ER‐α36 mRNA may serve as a good diagnostic biomarker for HCC. However, we barely detected ER‐α66 mRNA in the exosomes (data not shown). The exact reason for the low abundance of ER‐α66 in the exosomes from HCC patients needs to be further investigated.

In summary, ER‐α36 mRNA in the exosomes from HCC patients was significantly downregulated, indicating a possibility of using ER‐α36 mRNA in serum exosomes as a good diagnostic biomarker for HCC.

## CONFLICT OF INTEREST

The authors declare that the research was conducted in the absence of any commercial or financial relationships that could be construed as a potential conflict of interest.

## Supporting information

Supporting Information
