# Supplementary material for: Down‐regulation of ER‐α36 mRNA in serum exosomes of the patients with hepatocellular carcinoma
Source: Clin Transl Med. 2020 May 13;10(1):346–52. doi: 10.1002/ctm2.18 (PMC7240843; doi:10.1002/ctm2.18)
Supplement: Supplementary file 1 — Supporting Information [file CTM2-10-346-s001.doc]

**SUPPLEMENTAL TABLE**

**TABLE 1** The primer sequence of ER-α66 and ER-α36

|  | Forward primer | Reverse primer |
| --- | --- | --- |
| ER-α66 (NM_001122740.1) | 5’-GGTGCCCTACTACCTGGAGA-3’ | 5’-TCTGAATTTGGCCTGTAGAATG-3’ |
| ER-α36  (Bx640939) | 5’-GACAGGAACCAGGGAAAA-3’ | 5’-TCTACATGTGAGATACCAGA-3’ |
| GAPDH (NM_017008.3) | 5’-CTCCTCCACCTTTGACGCTG-3’ | 5’-TCCTCTTGTGCTCTTGCTGG-3’ |
